# Experimental investigation of the shear behavior at the pervious concrete-sand interface under monotonic loading

**DOI:** 10.1038/s41598-025-04075-4

**Published:** 2025-05-30

**Authors:** Mu’ath I. Abu Qamar, Ammar A. Alshannaq, Mohammad F. Tamimi, Motasem I. Al-Qura’an, Ghayda’a M. Alawneh

**Affiliations:** 1 Department of Civil Engineering, Yarmouk University, Irbid, 21163 Jordan; 2 Al-Amari Group, Irbid, Jordan; 3 Rami Zayadeen & Partners Contracting Company, Amman, Jordan

**Keywords:** Sand-pervious concrete interface, Sand-concrete interface, Surface roughness, Interface shear testing, Pervious concrete, Modified direct shear test device, Civil engineering, Structural materials

## Abstract

Interaction between soil and structural materials plays a critical role in the overall stability of geotechnical systems such as piles, retaining walls, soil nails, and soil anchors. Pervious concrete is increasingly being used as an alternative for conventional concrete in applications such as evaporative or wet cooling, ground improvement using microbial-induced carbonate precipitation (MICP) biogrouting, or possibly geothermal foundations (energy piles). The motivation and the aim of the present experimental study is to improve the understanding of shear behavior at the interface between pervious concrete and cohesionless soil under mechanical loading. This paper presents results from a series of interface direct shear tests performed on smooth, conventional concrete, pervious concrete of variable surface roughness sheared against fine and medium sands. It was found that the surface roughness of tested surfaces has a remarkable influence on the shear strength and volume change responses at sand-concrete interfaces. In the sand-pervious concrete tests, the interface shear strength and soil dilation increase as the surface roughness increases, which was significantly influenced by the porosity of concrete specimens. The smooth (untextured) interface exhibited a soil contractive behavior and yielded lower interface shear resistance among all surfaces, which is considered as the lower bound of strength. The results presented show that pervious concrete mobilizes interface shear strength and volume change increases with specimen porosity (i.e., 15% vs. 30%) and confining (normal) stress, which yielded a value ranges between 2.82 and 3.46 times of sand-smooth (untextured) surface strength in medium and fine sands, respectively.

## Introduction

The interaction between geotechnical systems (e.g., piles, retaining walls, soil anchors, and soil nails) and surrounding soils plays a crucial role in the overall stability and load-carrying capacity of these systems. The design and analysis of geotechnical systems require a comprehensive understanding of the interactions between soil and structural materials. Pervious Concrete (PC) is a type of concrete, fabricated with poorly-graded coarse aggregate with little to no fine aggregate which results in a volume with interconnected voids^1–4^. PC is widely known as sustainable permeable pavement materials to handle stormwater as well as to replenish groundwater and collect surface pollutants^1^. Pervious pavements helps in reducing noise, enhancing skid resistance, and reducing the urban heat island effect^5^. The porosity (i.e., void ratio) plays an important role on the mechanical properties (i.e., compressive and split-tensile strength) and permeability of pervious concrete. The permeability of pervious concrete is also influenced by aggregate surface area, void radius, tortuosity, and the volume of aggregate to paste ratio. It is widely accepted that the water permeability of pervious concrete increases as the porosity increases (or volume of paste decreases)^6–11^. Researchers have studied the use of pervious concrete for ground improvement applications^12^. For example, Lin et al.^12^ presented an innovative grouted ground improvement pile alternative (namely biogrouted pervious concrete pile). The biogrouting, as introduced in the research study, is a technique uses soil bacteria to induce calcium carbonate (CaCO_3_) to cement soil particles in vicinity of the pile. The research study evaluated the feasibility of microbial-induced carbonate precipitation (MICP) biogrouting of a limited zone of soil surrounding the pile to enhance the axial resistance of pile under compressive loads. Due to the inherent porosity of pervious concrete, the pervious concrete pile was used as an injection point during the MICP biogrouting^12^. However, the influence of pervious concrete pile roughness before and after MICP biogrouting has not been experimentally investigated by any research study. Researchers have explored and evaluated feasibility of the use of pervious concrete for evaporate or wet dry cooling applications^13–15^. Recent research studies examined feasibility of utilizing pervious concrete made with lightweight aggregate impregnated with phase change materials (PCM) for heating and cooling applications^10,11^. The studies also included examining the influence of PCM impregnation on the mechanical properties of pervious concrete made with lightweight aggregates. The use of lightweight aggregates in energy storage applications is preferred due to its inherent porous structure. The concept of pervious concrete piles with or without PCM could be extended to be used for heat exchange applications, in which pervious concrete energy piles could be used not only to exchange heat but also to resist structural loads. Recently, Zhang et al.^16^ studied the behavior of magnesia MgO-carbonated composite (MCP) pile using a modified direct shear test device, the proposed MCP pile consists of a pervious concrete pile (inner core) placed at the center of a deep magnesia (MgO) mixing column (outer column) under forced carbonation. As suggested by Zhang et al.^16^, the shaft resistance of the MCP pile is mainly governed by the pervious concrete-carbonated soil interface. The study focused on the influence of key governing factors such as carbonation time, MgO content, and initial water on the friction capacity at the interface, however, the surface condition (or roughness) or the design of pervious concrete were not considered as controlling factors. Therefore, the present study aims to consider the influence of pervious concrete surface roughness and its mix design on the shear behavior at the interface with cohesionless soils. To the best of the authors’ knowledge, the influence of surface roughness on the shear behavior at the interface between soil and pervious concrete has not been experimentally investigated either under mechanical or thermal loading.

This paper presents an experimental study on the shear behavior at the interface between pervious concrete and cohesionless soil, pervious concrete piles (permeable concrete piles) were previously utilized to improve the performance of the foundation systems by increasing shaft and/or tip resistance. For example, bio-grouting utilizes soil bacteria to induce calcium carbonate precipitation to cement cohesionless soil particles to improve its strength, stiffness, and dilatancy^12,17^. Moreover, Zhang et al.^16^ examined the use of pervious concrete in ground improvement systems. However, the influence of surface roughness on soil-pervious concrete interface shear behavior has not been experimentally investigated.

Numerous research studies with a focus on the mechanical response of soil-structural materials interfaces have been performed utilizing modifications to the standard interface testing devices, namely direct shear, simple shear, and ring shear^18–22^ and non-standard interface devices^23–26^ over the past decades. The role of several factors including surface roughness and hardness, confining (normal) stress, relative density, and particle size and shape on behavior of sand-structure interfaces have been extensively studied^27–33^. The load transfer mechanism between the soil and geo-structure surfaces occurs through friction and/or passive resistance^34,35^. Smooth to very low random roughness level surfaces (e.g., conventional concrete, rusted steel) transfer applied loads through friction, while medium to high roughness random, ribbed or structured surfaces (e.g., textured, pervious concrete) can mobilize additional interface shear resistance due to local shear and particle displacement (or additional resistance from entrapped soil or passive wedges) in addition to the interface friction^35–38^. Based on previous studies^32,39^, it was concluded that the interface shear resistance of random surfaces increases with the normalized roughness (R_n_) up to a critical roughness at which the interface shear resistance becomes constant. The normalized surface roughness (R_max_/D_50_) is the ratio of maximum roughness to the mean particle diameter (D_50_) of the tested sand. Previous research studies have investigated the shear behavior of soil-concrete interfaces under monotonic and cyclic loading^40–46^. For example, Kang et al.^42^ studied the friction characteristics of the interface between crushed rock with different roughness and conventional concrete through conducting monotonic and cyclic interface direct shear tests. Hossain and Yin^41^ conducted a series of interface direct shear tests between compacted, completely decomposed rock and cement grout under saturated condition at different normal stresses and grouting pressures. Chen et al.^40^ utilized a large-scale modified direct shear apparatus to examine the effect of surface roughness on the interfacial shear strength and shear behavior of red clay-concrete surface interfaces under monotonic loading. The test results suggested that the shear strength and shear dilation of red clay-concrete interface increases as the roughness of the concrete increases. Nardelli et al.^43^ examined the role of surface texture and confinement condition on the shear response of sand-concrete interface under monotonic loading. The study included performing a series of interface direct shear tests on concrete surfaces that simulate smooth-driven piles, rough-grouted piles, and rough-wavy screw piles under constant normal load (CNL) and constant normal stiffness (CNS) confinement conditions. Few research studies have focused on the influence of surface roughness on the shear behavior of soil-concrete surface interfaces under cyclic loading. For instance, Wang et al.^44^ conducted an experimental study on rough concrete block interfaces in red clay, the interface was subjected to cyclic loading using a large-scale direct shear test device. The study evaluated impact of surface roughness level, confining pressure applied on red clay, and number of load cycles on cyclic and post-cyclic shear strength and soil volume change. Wu et al.^45^ examined the effect of soil unloading and surface roughness on the shear behavior of sand-concrete interfaces through conducting a series of interface direct shear tests on concrete blocks that simulate the roughness condition of bored piles. Zhang et al.^46^ performed a series of interface direct shear tests on concrete blocks with irregular roughness under dynamic normal loading considering the influence of load amplitude and frequency, the study also included establishing discrete element method (DEM) models to analyze the mechanical behavior at microscopic scale. It is widely accepted by several researchers that the structural material’s (e.g., concrete) surface is highly irregular due to its inherent characteristics^46,47^, however, some researchers tend to assume that the surface roughness of structural materials surface is regular^48^.

The motivation and the aim of the present experimental study is to improve understanding of shear behavior at the interface between previous concrete of variable roughness and uniform size cohesionless soils, which provides insights toward utilizing the pervious concrete in engineered geotechnical applications such as biogrouted pervious concrete piles and pervious concrete energy piles. This paper summarizes the results of a series of interface direct shear tests performed on surfaces including smooth (untextured), conventional concrete, and pervious concrete in contact with uniform size cohesionless soil specimens. In addition to evaluating the interface shear response, the volumetric change response was also considered. The influence of surface roughness level and confining (normal) stress on the interface response was also evaluated and discussed.

## Materials and methods

### Sand

**Fig. 1:**
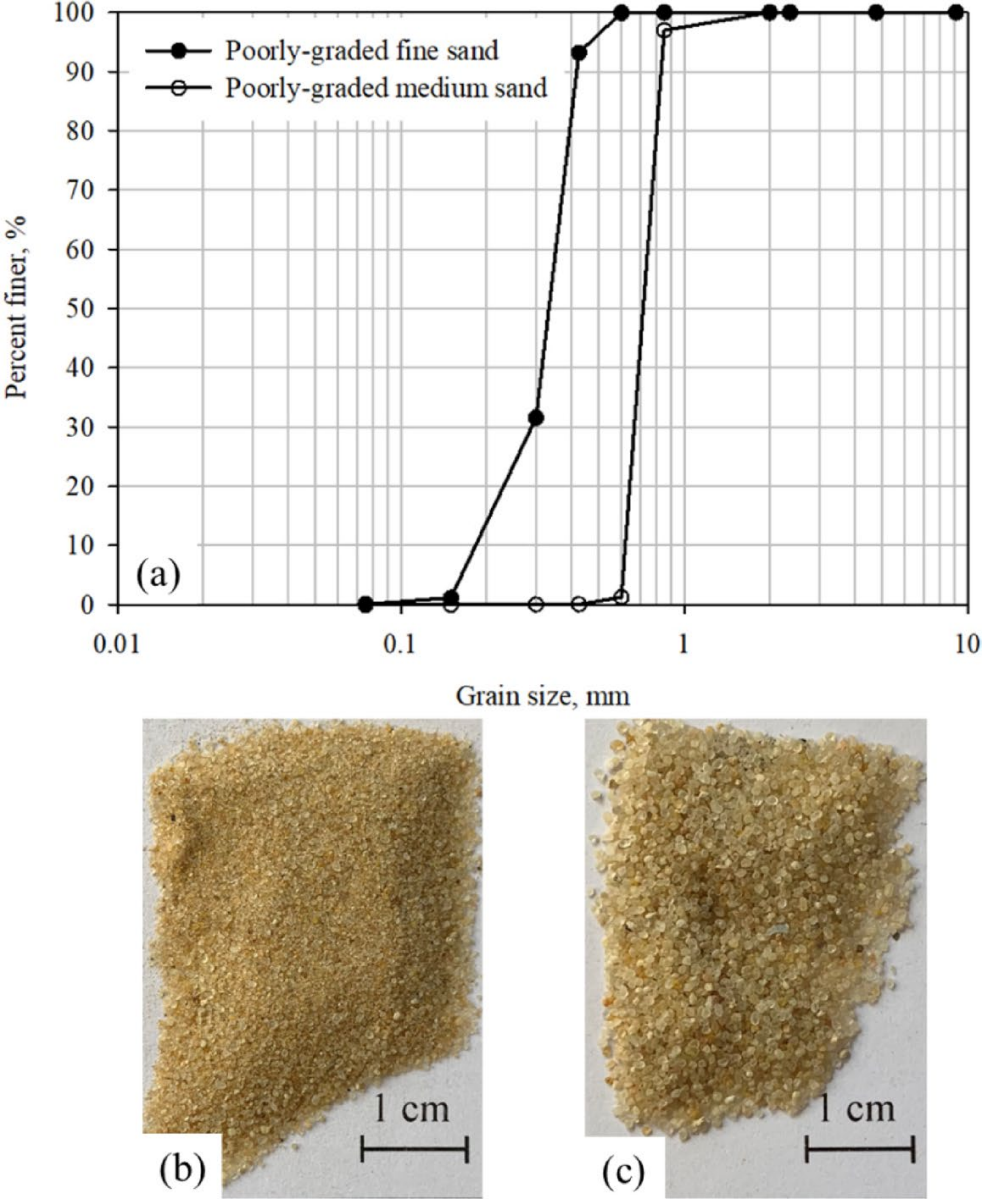
(**a**) Gradation curves for sands used in this study, (**b**) actual photograph of poorly-graded fine sand, and (**c**) actual photograph of poorly-graded medium sand.

A locally available silica sand (obtained from a construction site in Irbid, Jordan) was used as a base material in the present study. This sand is a mixture of fine and medium sands and is classified as poorly-graded sand (i.e., SP). The poorly-graded sands were selected to simulate soils with large pore throats that allow the easy movement of microbials before the precipitation of CaCo_3_ when MICP bio-grouting pervious concrete piles are used to enhance the response of ground improvement and foundation systems^12,17,49,50^. To produce sands with uniform particle sizes, two different sizes were extracted from the original sand. The uniform size sands were tested in this investigation to study the shear response and behavior of pervious concrete (PC) in comparison to conventional concrete as well as smooth (untextured) surfaces under monotonic loading. According to the Unified Soil Classification System (USCS)^51^, the extracted sands are poorly-graded fine and medium sands with coefficient of uniformity (*C*_u_ = *D*_60_/*D*_10_) of 1.89, 1.16 and coefficient of curvature (*C*_c_ = (*D*_30_)^2^ /*D*_60_ × *D*_10_) of 1.28, 0.95, respectively. The grain size distribution (GSD) curves and actual photographs of the extracted poorly-graded fine and medium sands are shown in Fig. 1. The poorly-graded fine and medium sands have a mean particle diameters (*D*_50_) of 0.32 and 0.70 mm, respectively. Table 1 summarizes the characteristics of both sands including maximum and minimum void ratios, coefficient of uniformity and coefficient of curvature, mean particle size, sand solid’s specific gravity, and internal angle of friction.

**Table 1:** Characteristics of the poorly-graded sands used in present study.

Parameter	Poorly-graded fine sand	Poorly-graded medium sand
Coefficient of uniformity (*C*_u_)	1.89	1.16
Coefficient of curvature (*C*_c_)	1.28	0.95
Median particle size (*D*_50_) – (mm)	0.32	0.70
Specific gravity (*G*_s_)	2.58	2.61
Maximum void ratio (*e*_max_)^1^	0.87	0.88
Minimum void ratio (*e*_min_)^2^	0.56	0.57
Internal angle of friction (*ϕ*)^3^ – (°)	34.9	37.9

^1^ASTM D4254–16^52^, ^2^ASTM D4253–16^53^, ^3^conventional direct shear tests^58^.

**Fig. 2:**
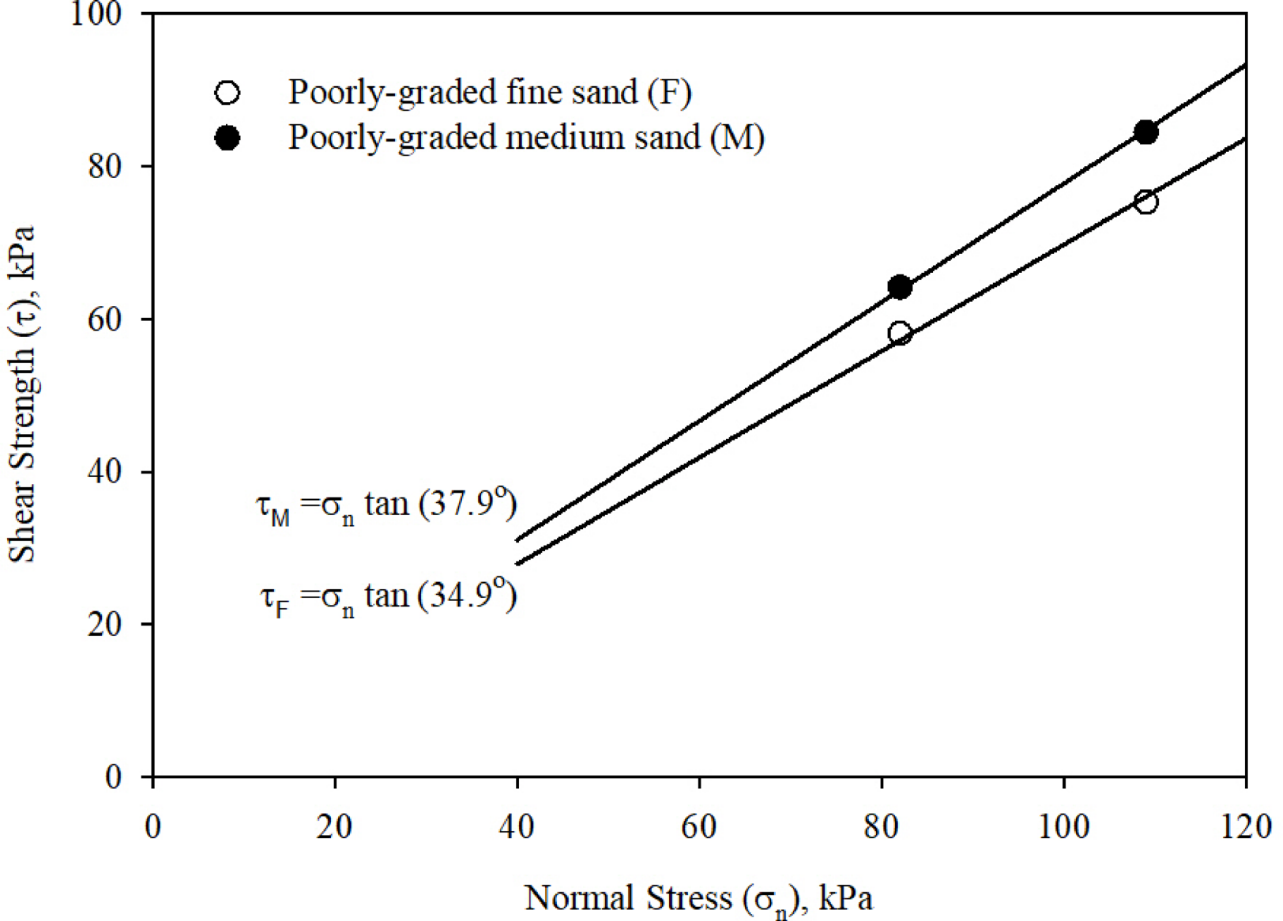
Strengh faliure envelopes for poorly-graded fine and poorly-graded medium sands using conventional direct shear apparatus.

To determine the shear strength parameters (i.e., internal angle of friction (*ϕ*)) of the two sands, a series of conventional direct shear tests were performed on representative sand specimens using a conventional shear box. All specimens were air-pluviated in the box to a target relative density (*D*_r_) of 80 ± 1%, this was maintained for all conventional and interface direct shear tests. The specimens were 60 mm in length, 60 mm in width, and 35 to 37 mm in height. The results of the conventional direct shear tests were used to develop Mohr-Coulomb (MC) strength envelopes for both the poorly-graded fine and the poorly-graded medium sands. The strength envelopes are presented in Fig. 2. As shown in Fig. 2; Table 1, the internal angle of friction (*ϕ*) of fine and medium sands are 34.9° and 37.9°, respectively.

### Concrete

As mentioned previously, this work investigates the shear response and behavior of the interface between smooth (untextured), conventional concrete, and pervious concrete of different porosity tested against fine and medium sands. Thus, a locally available sand that has mean particle size (*D*_50_) and soil solid’s specific gravity (*G*_s_) of 0.58 mm and 2.64, respectively, was used as fine aggregate in fabricating the conventional concrete blocks used in the present study. The concrete blocks used herein consist of cement (C), poorly-graded sand (S), and water (W) in the ratio by weight of 1:2.75:0.6, respectively. The water to cement (*w*/*c*) ratio of the conventional concrete mix was 0.6.

**Fig. 3:**
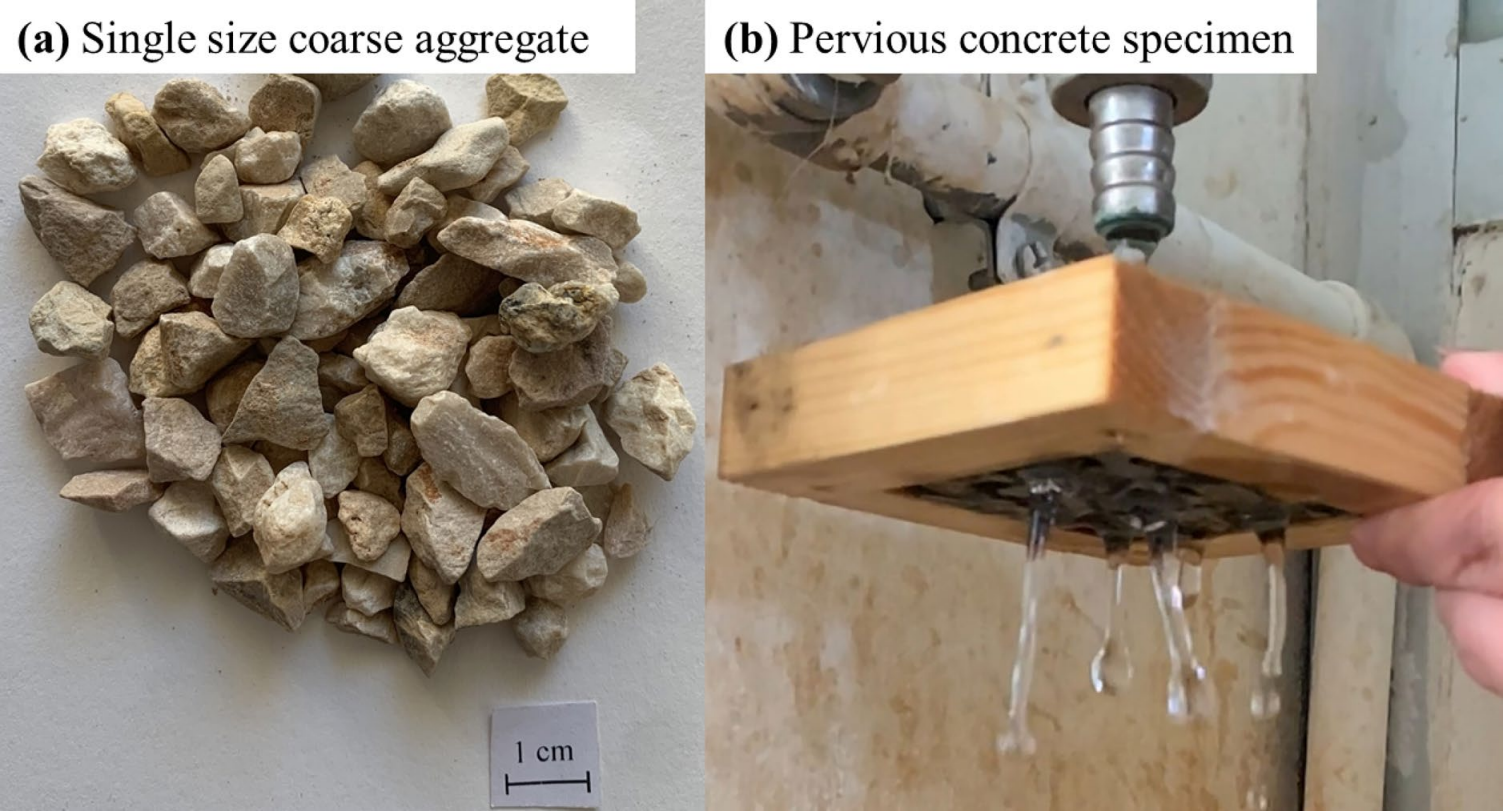
(**a**) Actual photograph of the single size coarse aggregate used to fabricate concrete specimens; and (**b**) Actual photograph of pervious concrete specimen under water source.

Moreover, to produce pervious concrete specimens, a locally available normal weight aggregate was used as shown in Fig. 3a. The aggregate used to fabricate pervious concrete blocks consists of a narrow range particle size with 100% passing through the 9.5 mm (3/8 in.) opening size sieve and 100% retained on the 4.76 mm (No. 4) opening size sieve. The capacity of aggregate to absorb water was determined through immersion of oven dry representative specimens in water for 24 h, the water absorption was measured in accordance with the ASTM C127-12^54^. Table 2 summarizes the physical properties of coarse normal weight aggregates. Based on the fact that pervious concrete is a type of concrete that is made with single size coarse aggregate and little or no sand, therefore, the aggregate with a narrow range of particle size was selected to possess a better control on the porosity of pervious concrete^1–4^. The porosity as well as the permeability are significantly influenced by the existence of aggregate of wide range of sizes or fine sand in the mixture^10,11^.

**Table 2:** Physical properties of normal weight aggregate used in present study.

Parameter	Value
Unit weight (kg/m^3^)	1525
Void content (%)	40.98
Specific gravity	2.59
Water absorption (%) by mass	3.3

The pervious concrete specimens used to evaluate the interface shear resistance between fine and medium sand was designed in accordance with the approach proposed by the National Ready-Mix Concrete Association (NRMCA)^55^. Design parameters such total porosity (*P*), water to cement (*w*/*c*) ratio, and dosage of high range water reducing (HRWR) admixture have a significant influence on the fresh and hardened properties of pervious concrete. As suggested by the ACI Committee 522^1^, pervious concrete made with normal weight aggregate has a total porosity in the range between 15 and 35%, water to cement (*w*/*c*) ratio in the range between 0.27 and 0.43, and coefficient of water permeability in the range between 0.1 and 1.2 m/s. The porosity of pervious concrete has lower and upper bounds, where concrete with approximately zero porosity corresponds to conventional concrete (i.e., lower bound) and concrete with relatively higher porosity close to aggregate void ratio corresponds to bare aggregates (i.e., upper bound). Similarly, the porosity of concrete conversely influences the compressive strength, where pervious concrete with low porosity yields higher compressive strength than concrete with higher porosity. As the porosity of pervious concrete impacts the water permeability, it also influences the surface roughness of the produced concrete. During preliminary testing, pervious concrete specimens were prepared at different porosity (or void content) of 15, 20, 25, 30, 35%. As observed, lower porosity could influence surface roughness, which might impact the frictional resistance of concrete elements in contact with soil. Conversely, higher porosity significantly influences the surface roughness, which may enhance the frictional resistance of concrete elements in contact with soil. Therefore, the porosity of concrete was varied to investigate the shear behavior at the interface between pervious concrete and sandy soil in comparison to smooth (untextured) surface that simulates the surface condition of smooth steel pipe piles.

### Pervious concrete mix design and porosity measurements

The approach proposed by the National Ready-Mix Concrete Association (NRMCA)^55^ was adopted to prepare specimens with different porosity, thus, different surface roughness. Type II Portland cement is utilized in this investigation with water to cement (*w*/*c*) ratio of 0.30 and a porosity (*P*) of 0%, 15 ± 1%, and 30 ± 1%. A series of trial pervious concrete mixes were fabricated with the locally available normal weight aggregate (see Table 2), the aggregate used to fabricate pervious concrete specimens consists of a narrow range particle size (see Fig. 3a). An actual photograph of a pervious concrete with proofed porosity under a water source is shown in Fig. 3b.

**Table 3:** Mix proportions and porosity of pervious concrete specimens used in present study.

Mix label	Cement (kg/m^3^)	Aggregate (kg/m^3^)	Water (kg/m^3^)	Sand (kg/m^3^)	Porosity (%)
Pervious concrete (Porosity = 15%)	501	1398	150	0	14.9
Pervious concrete (Porosity = 30%)	259	1398	78	0	29.9

To fabricate pervious concrete specimens for porosity measurements and interface shear testing, the mixing procedure reported by Kevern et al.^56^ was adopted. A consistent procedure was used to produced aggregates with same initial conditions, it consists of the following steps: (1) soaking (or saturating) the single size aggregate for 24 h to ensure that all empty pores are filled with water prior to concrete fabrication (despite of the low water absorption); (2) draining the excess water out of the aggregate for four hours; and (3) measuring the moisture content prior to batching to account for the surface water held be the aggregate which allows for the adjustment of the water content. All pervious concrete specimens were fabricated using a consistent procedure that consists of the following steps: (1) mixing the saturated coarse aggregates with 5% of the total cement content using a bench top auto mortar mixer for a duration of 1 min; (2) adding and mixing all remining ingredients for three minutes; (3) allowing the final mix was to rest for two minutes, followed by a final three minutes mixing was done; and (4) casting concrete in the molds as shown in Fig. 3b.

The porosity of the hardened pervious concrete specimens was determined in accordance with the ASTM C1754-12^57^. To determine the porosity, cuboid specimens that are 60 mm in length, 60 mm in depth, and 20 mm in height were used. Three representative specimens from each mix were cured in water at room temperature ranging from 22 to 23 °C overnight, the specimens were then weighed in water. After taking the weight of the specimens in water, the specimens were placed in the oven at a temperature between 105 °C and 110 °C for 24 h. The dry weight and volume of each specimen were obtained. The porosity (*P*) of the representative specimens is calculated as the difference between the dry weight and weight in water as shown in Eq. (1).

1 $$P = ~\left[ {1 - \left( {\frac{{W_{d} - W_{w} }}{{\rho _{w} ~.~V_{s} }}} \right)} \right] \times 100\%$$

In Eq. (1), *P* is the porosity (%)(percentage of voids to total volume of specimen); *W*_d_ is the dry mass of specimen after 24 h in oven (g); *W*_w_ is the weight of specimen in water (g); *V*_s_ is the total measured volume of each specimen (cm^3^); and *ρ*_w_ is the water density (g/cm^3^). As the National Ready-Mix Concrete Association (NRMCA)^55^ mix design approach does not account for shape and surface characteristics of the aggregates and the cement paste thixotropy, a marginal difference between design and measured values was observed. The results of the measured porosity of fabricated mixes are summarized in Table 3.

### Test surface design and geometry

During preliminary tests, It was observed that the surface roughness of a pervious concrete specimen increases as the volume of paste to bond aggregate decreases (or porosity increases), which allows varying its surface roughness, however, increasing porosity will lead to a significant reduction in the compressive strength of pervious concrete^3^. A total of four surfaces were tested as part of the present study. All surfaces are 60 mm in length, 60 mm in width, and 18 to 20 mm in thickness. The surfaces are as follows: (1) smooth (untextured) surface; (2) conventional concrete block (resulted from zero porosity condition); (3) rough pervious concrete block (resulted from 15% porosity condition); and (4) rough pervious concrete block (resulted from 30% porosity). The smooth (untextured) surface was made with aluminum in the engineering workshop unit at Yarmouk University, the surface was treated to achieve a high level of smoothness. The smooth surface was used to simulate the surface condition of commonly used smooth steel piles. The conventional concrete surface simulates the roughness condition of a precast pile as a foundation element supports imposed loads. The pervious concrete surfaces with 15 and 30% porosity conditions simulate the surface roughness condition of a pervious concrete pile with MICP bio-grouting as ground improvement technique^12^. To perform interface shear tests on conventional concrete and pervious concrete, two representative specimens from each mixture were prepared by casting them into wooden molds.

**Fig. 4:**
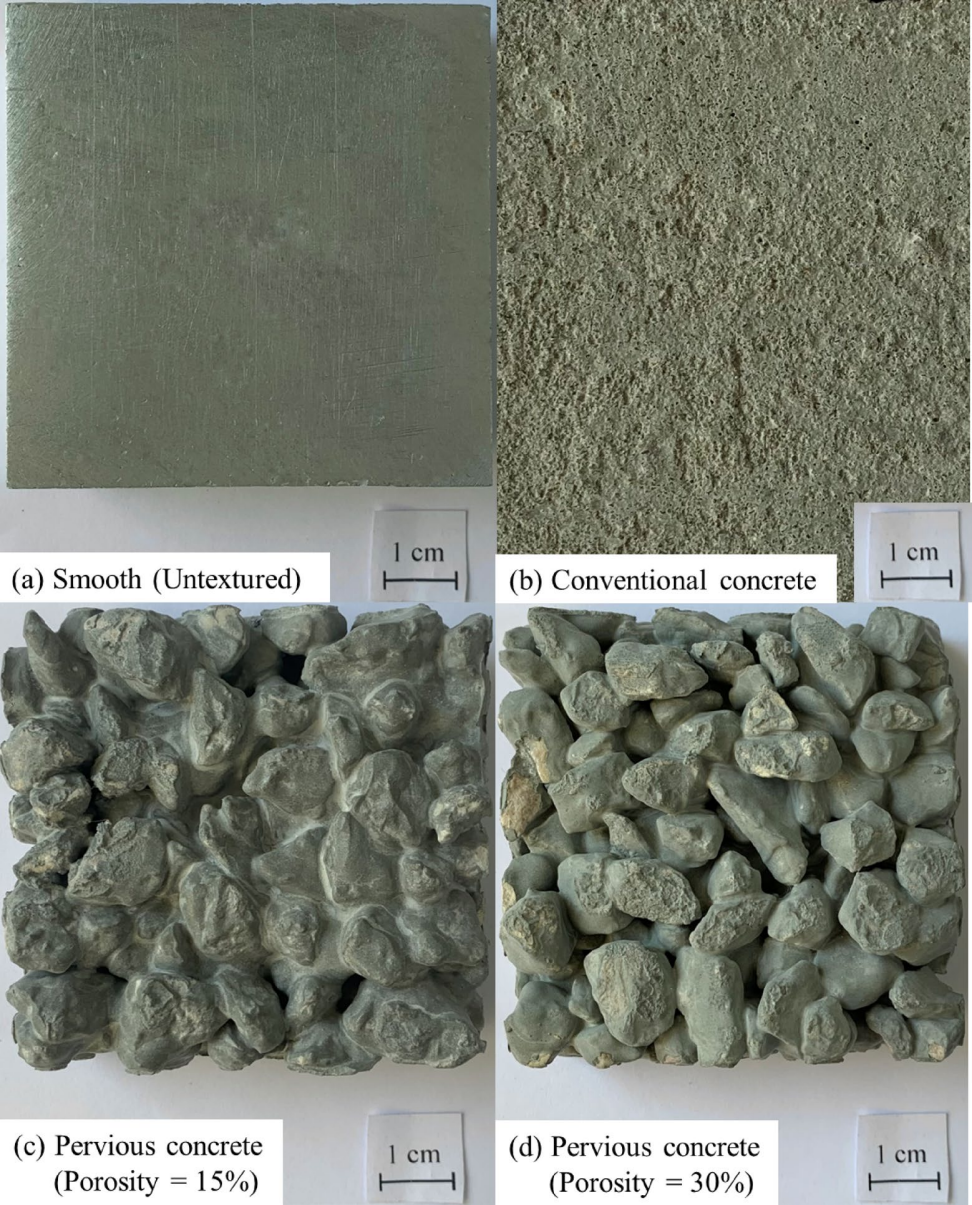
Actual photographs of the surfaces used in this study: (**a**) smooth (untextured); (**b**) conventional concrete; (**c**) pervious concrete with 15% porosity; and (**d**) pervious concrete with 30% porosity.

Actual photographs of the smooth, conventional and pervious concrete blocks are shown in Fig. 4. As shown in Fig. 4, unlike smooth and conventional concrete blocks, the roughness of the prepared pervious concrete blocks is random due to spatial distribution of coarse aggregates within the volume of the block, which is mainly influenced by the design porosity, aggregate size and shape, and water to cement ratio.

Interface tests against these surfaces (Fig. 4a–d) were conducted to investigate the effect of surface roughness on the shear strength and volume change in poorly-graded fine and medium sand. During an interface test, the sand specimen was rained (air-pluviated) over the surface, followed by the application of confining (normal) stress and displacing the surface till failure. It is important to note that the thickness of the molds used to prepare the smooth and concrete blocks equal to the bottom half of the shear box to minimize the effects of boundaries on the measured responses. The modification of the conventional shear box will be discussed in detail in the next sections. Additional tests were performed on all surfaces to investigate the effect of confinement level on the interface shear strength and volume change in poorly-graded fine and medium sands.

## Experimental setup

### Interface shear testing setup

**Fig. 5:**
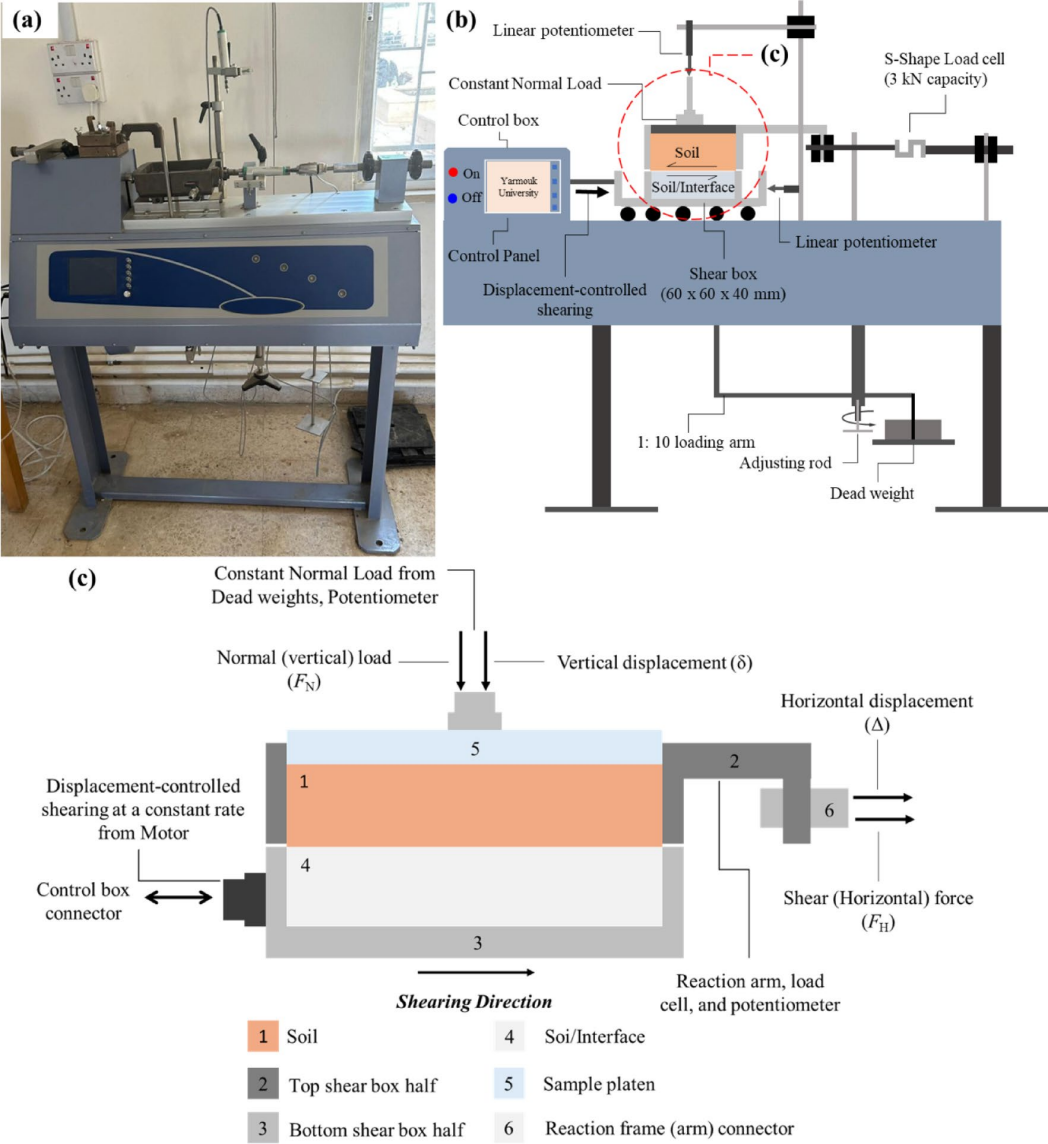
(**a**) An actual photograph of the direct shear used in this study; (**b**) Conventional/Modified direct shear test apparatus with all components; and (**c**) Schematic of the shear box, showing all measured responses, including shear force, shear displacement, soil volume change, and the direction of shearing.

The Digital Shear Testing Machine, (S277-01, MATEST, Italy), was modified to perform interface shear testing by replacing the conventional bottom half of shear box with different surfaces connected to the moving sled. The conventional/modified direct shear test apparatus as well as an actual photograph of the apparatus are shown in Fig. 5a,b. The interface testing system consists of a shear box assembly, loading (reaction) arm, and a control box. The shear box consists of two halves, the top half is rigidly connected to the loading arm and the bottom half is fastened to the sled. The load (reaction) arm allows the friction force transfer at the soil-surface and soil-soil interface to the horizontal S-shape load cell. The system allows the application of normal load to the specimen using a loading arm with a 1:10 ratio, it also includes an automatic motor that applies a shear force on the interface between soil specimen and soil-surface in direct shear and interface shear tests as shown in Fig. 5a,b. The apparatus motor (control box) allows for performing displacement-controlled conventional and interface direct shear tests at constant shearing rate in the range between 0.00001 mm/min and 10 mm/min.

The shear displacement (i.e., horizontal displacement (Δ)) that presents the relative movement between the two halves of the shear box and the soil volume change (i.e., vertical displacement (*δ*)) of soil specimen (either contraction or dilation) were monitored and measured using two linear potentiometers, the horizontal and vertical potentiometers have measuring ranges of 25.4 mm and 12.7 mm, respectively. As prescribed by ASTM D3080/D3080M^58^, the contact length (i.e., side length of the bottom half of the shear box) to the maximum particle size in the tested sands ratio should exceed 10. A length to particle diameter ratio of 73 is achieved with the configurations used in this study. Figure 5c shows a schematic view of the shear box, showing all measured responses, including normal force (*F*_N_), shear force (*F*_H_), shear displacement (Δ), soil volume change (*δ*), and the direction of shearing. The normal load (*F*_N_) applied to the specimen was back-calculated to achieve target confining (normal) stress, the shear load (*F*_H_) applied to the sand specimen was measured utilizing the load cell, the load cell has a tension-compression capacity of 3,000 N. The shear stress (*τ*) mobilized at the interface was determined as the measured shear force divided by the contact area between soil and material (i.e., cross-sectional area of specimen), and the stress ratio which was computed as the division of the shear stress mobilized at the interface by the applied confining (normal) stress imposed on the top boundary of the soil specimen. As suggested by Martinez et al.^59^, the sample platen was not constrained to prevent any possible rotation, thus ensuring that the applied confining (normal) stress was constant during the interface shear test stages. The modified apparatus allows performing interface shear tests under a constant normal load (CNL) confinement condition. The tests were performed at three different constant confining (normal) stresses of 50, 100, 150 kPa, which allows the sand specimen filling the top shear half to contract or dilate with shear displacement. The values of confining (normal) stress were selected to simulate the level of confinement experienced by foundation elements such as soil-pile system for most geotechnical applications. During all conventional and interface direct shear tests, the bottom half of the shear box was displaced at constant shearing rate of 0.5 mm/min (0.0083 mm/s). Tests were performed on poorly-graded fine and medium sands against smooth, conventional, pervious concrete surfaces under similar conditions (i.e., normal stress, relative density, shearing rate, and shearing direction) to allow for comparison between the measured responses.

**Fig. 6:**
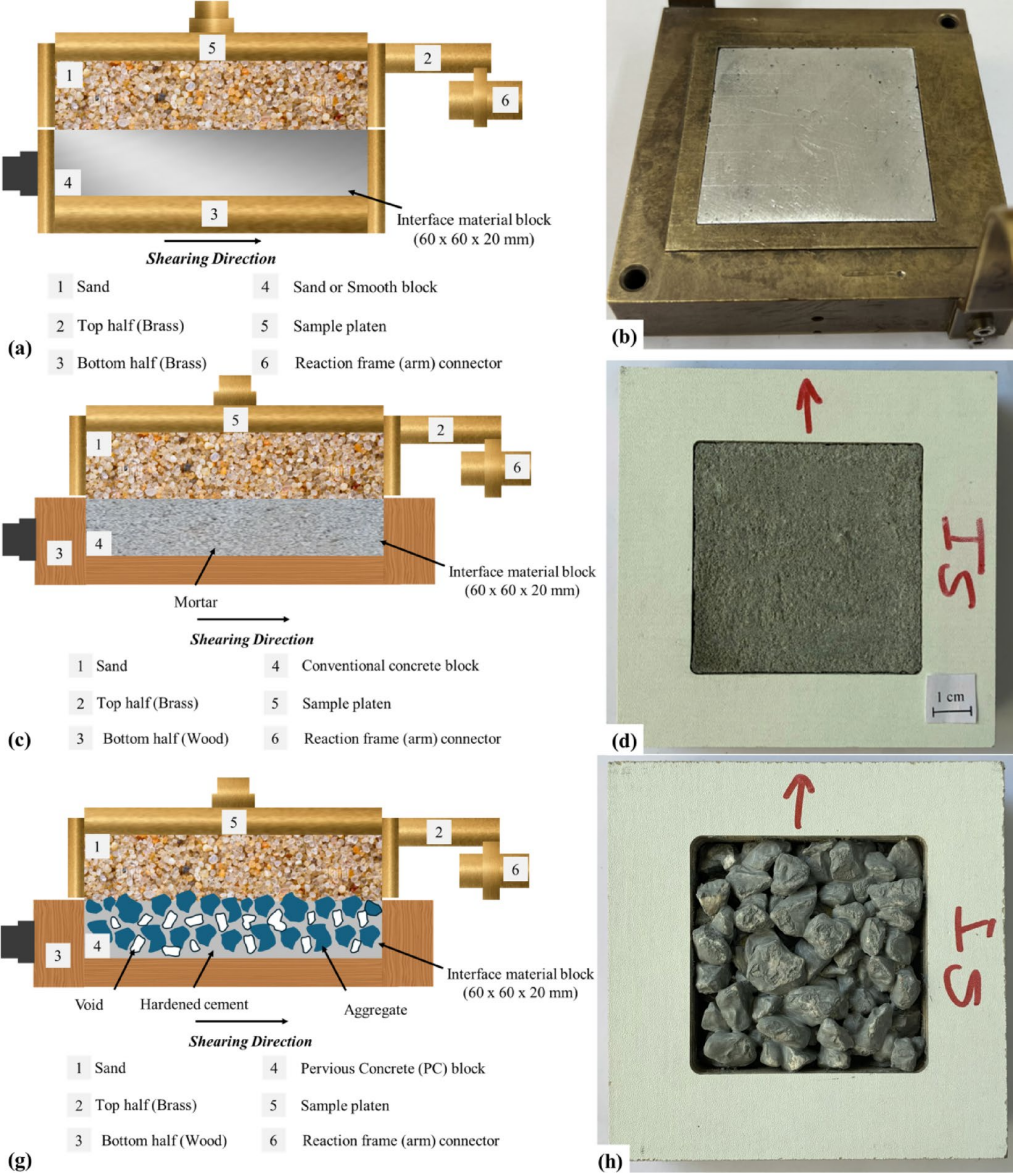
Interface shear testing configurations to evalute sand-surface strength: (**a** and **b**) conventional shear box with smooth block placed in the bottom half; (**c** and **d**) conventional top half with wooden bottom contains conventional concrete block; (**g** and **h**) conventional top half with wooden bottom contains pervious concrete block.

Figure 6 shows the configuration of the modified interface shear test system that was utilized to directly measure interface shear resistance of pervious concrete blocks with different surface roughness compared to smooth and conventional concrete blocks. Figure 6a,b show the details of the modified shear box and an actual photograph of the smooth surface block occupying the conventional bottom half of the shear box. To determine the interface shear resistance between sand and conventional and pervious concrete surfaces, the bottom brass (see Fig. 6b) half of the conventional shear box was replaced by a wooden cuboid that has external dimensions of 100 mm (length), 100 mm (width), and 23 mm (thickness) as shown in Fig. 6c,d. To produce concrete blocks with the same cross-sectional area, an empty volume of 60 mm × 60 × 20 mm was used to cast concrete for interface shear tests. The inner side of the empty volume (60 mm × 60 × 20 mm) was soaked in water (wetted) prior to concrete casting to avoid any loss of mixing water. The wet mixture of conventional concrete and pervious concrete of different porosity was placed and left to harden overnight at room temperature of 22 to 23 °C then cured in water to allow for the formation of cement hydrates and bonds. Preliminary work was conducted to ensure that the conventional concrete block has a level surface prior to interface testing. Similarly, the wooden cuboid was used to prepare pervious concrete specimens with different porosity levels, thus, blocks with different surface roughness. Figure 6g,h show the details of the shear box used to perform interface shear tests on pervious concrete in fine and medium sands and an actual photograph for a pervious concrete block prepared at a design porosity of 30%. Several research studies e.g^60–63^, have adopted the lower-plate shear setup to perform interface shear tests using modified direct shear test apparatus.

## Test results and discussion

All results reported herein are from monotonic tests to a shear (horizontal) displacement up to 4 mm, as the shear strength is fully mobilized. The positive sign volume change describes soil contraction while negative sign volume change indicates soil dilation.

### Interface shear strength of smooth surface

**Fig. 7:**
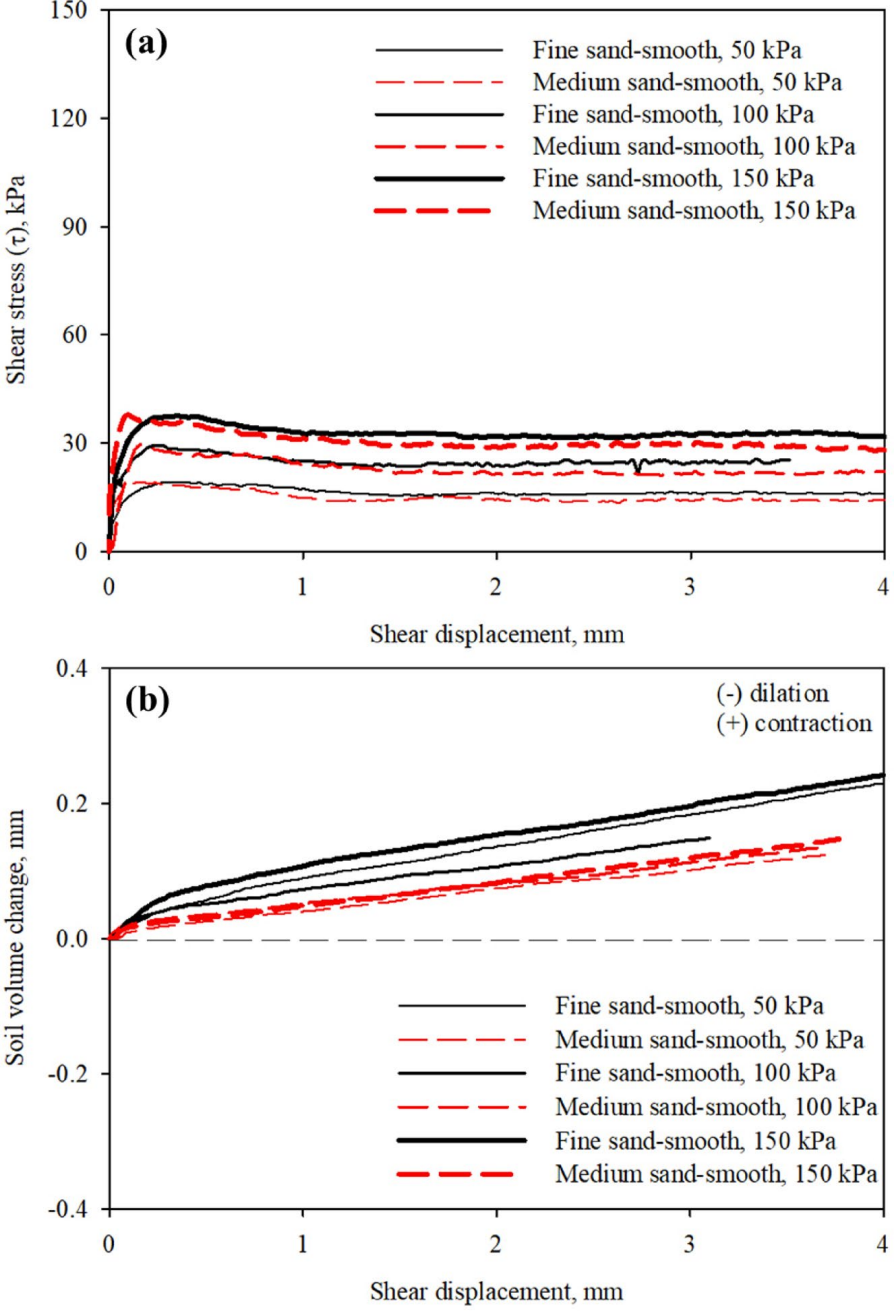
Resutls from interface tests on smooth (untextured) surface in fine and meidum sands: (**a**) mobilized shear stress with shear displacement; and (**b**) soil volume change with shear displacement, at 50, 100, 150 kPa confining (normal) stress.

Smooth (untextured) surface that simulate the surface condition of smooth steel piles, presented in Figs. 4 and 6, were tested to evaluate the interface shear resistance and soil volume change as a base (reference) response. Figure 7a,b show the results from these tests at different confining (normal) stresses. As shown in Fig. 7a, the mobilized shear stress at the interface between poorly-graded fine sand and poorly-graded medium sand increases as confining (normal) stress increases. Larger interface shear strength was mobilized at the fine sand-smooth surface interface than strength at the medium sand-smooth surface, which could be attributed to difference in the particle size of sand grains (mainly *D*_50_ of soil). As can be seen in Fig. 7a, all the shear stress-shear displacement exhibit a stick-slip behavior which is followed by softening, which was also observed by Fakharian^64^, Hamid and Miller^65^, and Chen et al.^40^, and Abu Qamar and Suleiman^66^.

The shear force transfer at the interface between sand and smooth (untextured) surface mainly occurs through friction, the shear force increases with the confining (normal) load and with the coefficient of friction as it represents the level of surface roughness. As the confining (normal) load applied to the soil specimen increases, the soil-surface contact area increases which results in increasing the shear strength at the soil-smooth interface. It can also be observed that the shear stress to confining (normal) stress ratio decreases with level of confinement, which is consistent with Chen et al.^40^. Figure 7b presents the soil volume change-shear displacement response curves from the tests on smooth surface at different confining (normal) stress values. It was observed that fine- and medium- smooth interfaces exhibited dominant contractive behavior under monotonic loading. The poorly-graded fine sand experienced larger volume contraction than the poorly-graded medium sand, also the amount soil contraction increases with shear displacement and level of confinement. As suggested by previous studies e.g^37,40,46,67^ smooth surfaces don’t develop shear zones at the interface with soil and the resistance to applied loads is characterized by friction.

### Interface shear strength of conventional rough concrete surface

**Fig. 8:**
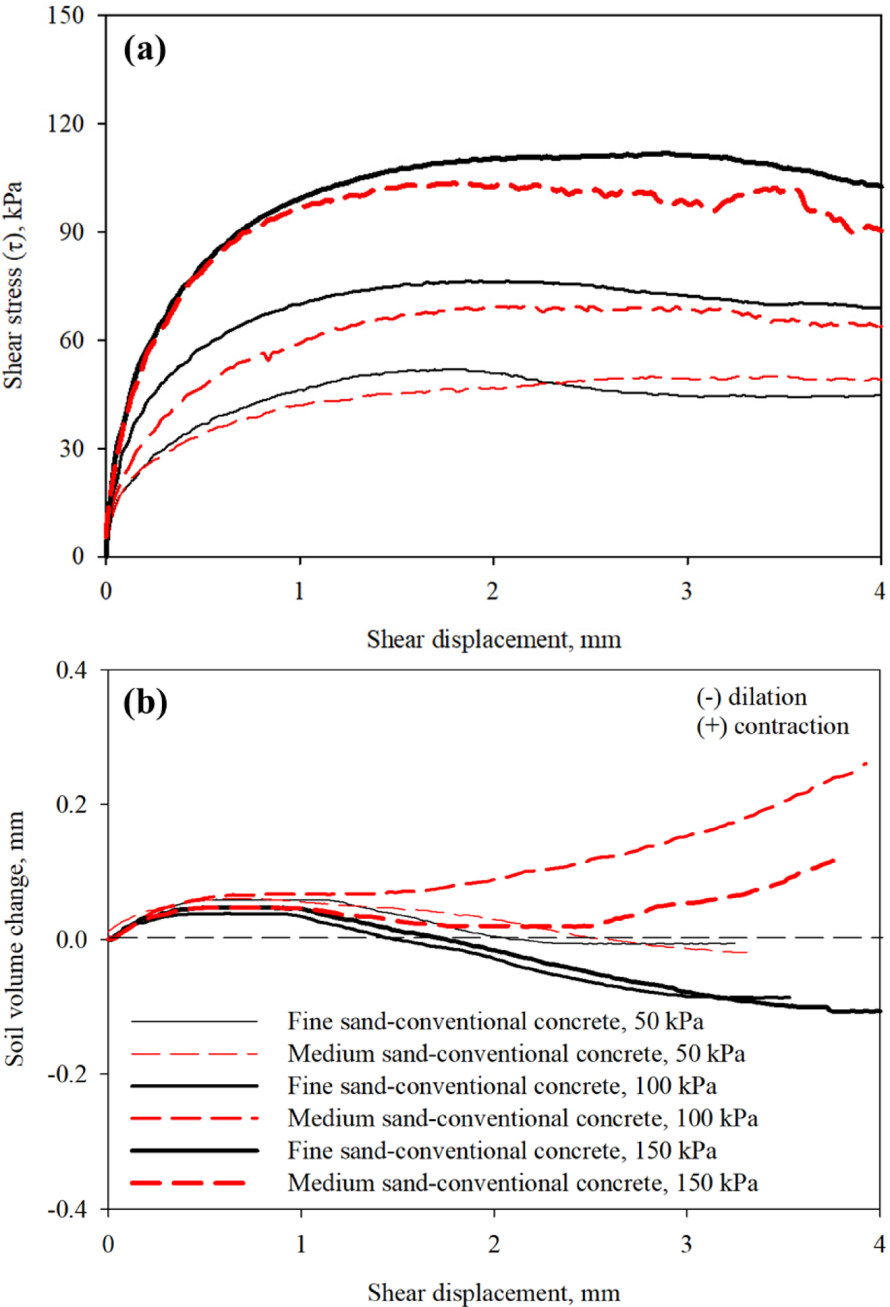
Results from interface shear tests on conventional concrete surface in fine and medium sands: (**a**) mobilized shear stress with shear displacement; and (**b**) soil volume change with shear displacement, at 50, 100, 150 kPa confining (normal) stress.

The roughness of surface plays an important role in the shear strength and stability of foundation elements under axial loading. As widely accepted by the research community^19,34,68^, shear load transfer at the interface between soil and surfaces with random roughness mainly occurs through interlocking and dilation of soil particles. Figure 8a,b present results from interface shear tests on conventional rough concrete surface in fine and medium sands prepared at target relative density of 80 ± 1%. The fine sand- and medium sand-conventional concrete interfaces at the lower confining (normal) stress of 50 kPa yielded shear resistance of 50 kPa and 45 kPa as shown in Fig. 8a. As can be observed in Fig. 8a, the shear resistance mobilized at the interface increased with confining (normal) stress. The medium sand-conventional rough surface interface exhibited a dominant contractive behavior, however, the fine sand-conventional rough surface interface experienced volume dilation with shear displacement, which results in larger interface shear resistance (see Fig. 8b).

Larger interface shear resistances at all confining (normal) stress levels were mobilized at sand-conventional concrete surface interface than sand-smooth interface shear resistance, the difference in the resistance is attributed to the difference in surface roughness as it increases as the roughness increases. The results reported herein are consistent with results from interface shear tests on sand-structural material surfaces reported by previous research studies e.g^40,43,45,46,69^. In the case of poorly-graded fine sand, the roughness of surface allows the particles to entrap in the asperities and causes soil dilation and shear failure within soil mass (i.e., soil-soil failure), which was observed by Uesugi and Kishida^32^. In the case of poorly-graded medium sand (i.e., coarser sand), the particle size is larger than the size of surface asperities which results in shear load transfer mechanism at soil-surface through sliding with a dominant contractive behavior, thus, smaller interface shear resistance.

### Interface shear strength of rough pervious concrete surfaces

**Fig. 9:**
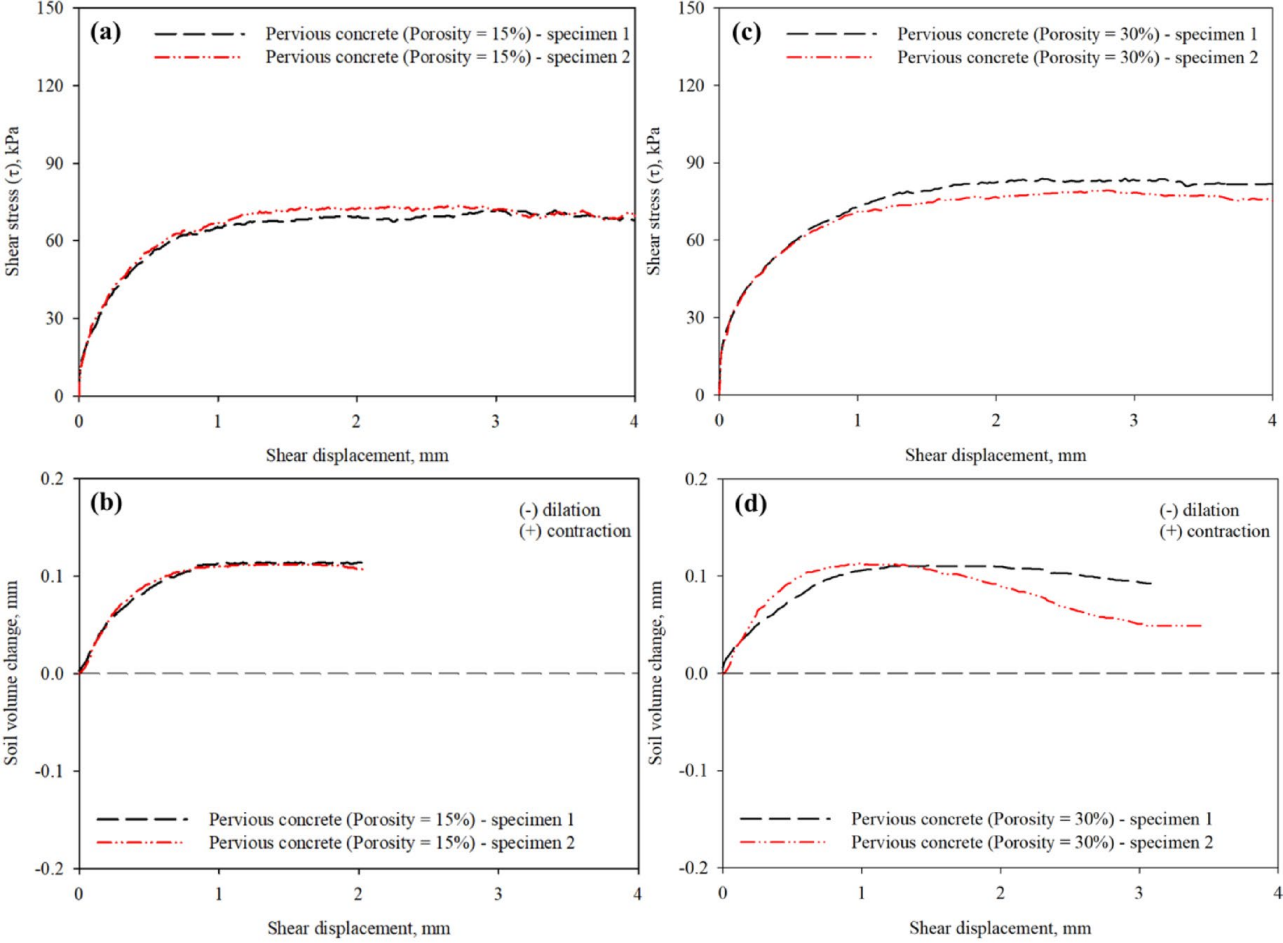
Results from interface tests on two different pervious concrete (PC) specimens of 15% and 30% porosity in poorly-graded medium sand: (**a** and **c**) mobilized shear stress with shear displacement, and (**b** and **d**) soil volume change with shear displacement, 100 kPa confining (normal) stress.

To the best of the authors’ knowledge, there are no experimental studies with a focus on evaluating influence of surface roughness on the shear response and volume change behavior at the interface of sand and pervious concrete piles. In the absence of preparation procedure that allows the control of pervious concrete pile surface roughness in the literature, the only mean to control the surface roughness is through varying design porosity of concrete specimens. As the design porosity increases, the roughness of pervious concrete surface increases (i.e., larger interconnected voids) due to the reduction in volume of paste that bond aggregates together. Preliminary tests were performed on pervious concrete specimens of same design porosity to examine the preparation procedure used in this study. Figure 9 presents results from the preliminary interface shear tests on pervious concrete specimens of different design porosity (labeled as specimen 1 and specimen 2) sheared against sandy soil under 100 kPa confining (normal) stress. Figure 9a,b shows the mobilized shear stress-shear displacement and soil volume change-shear displacement response curves for the specimens with 15% porosity. In the case of 15% porosity, response curves for both specimens are in good agreement and show marginal difference. The observed marginal difference could be attributed to the cement paste volume being sufficient to coat the coarse aggregate and produce concrete surface with consistent roughness. Figure 9c,d shows the mobilized shear stress-shear displacement and soil volume change-shear displacement response curves for the specimens with 30% porosity. In the case of 30% porosity, response curves for both specimens are in good agreement and show marginal difference up to 1 mm shear displacement. A more significant difference is observed between 1 and 4 mm in both shear stress-shear displacement and soil volume change-shear displacement response curves. The observed difference could be attributed to the cement paste volume not being comparably sufficient such that it completely coats the coarse aggregate and produces concrete surface with consistent roughness, which results in roughness irregularity, thus, difference in the response. This is inevitable due to the large void ratio of 30%. Therefore, with this observed marginal difference in results, the preparation procedure could be adopted to evaluate the shear behavior and volumetric response of pervious concrete in sandy soils.

**Fig. 10:**
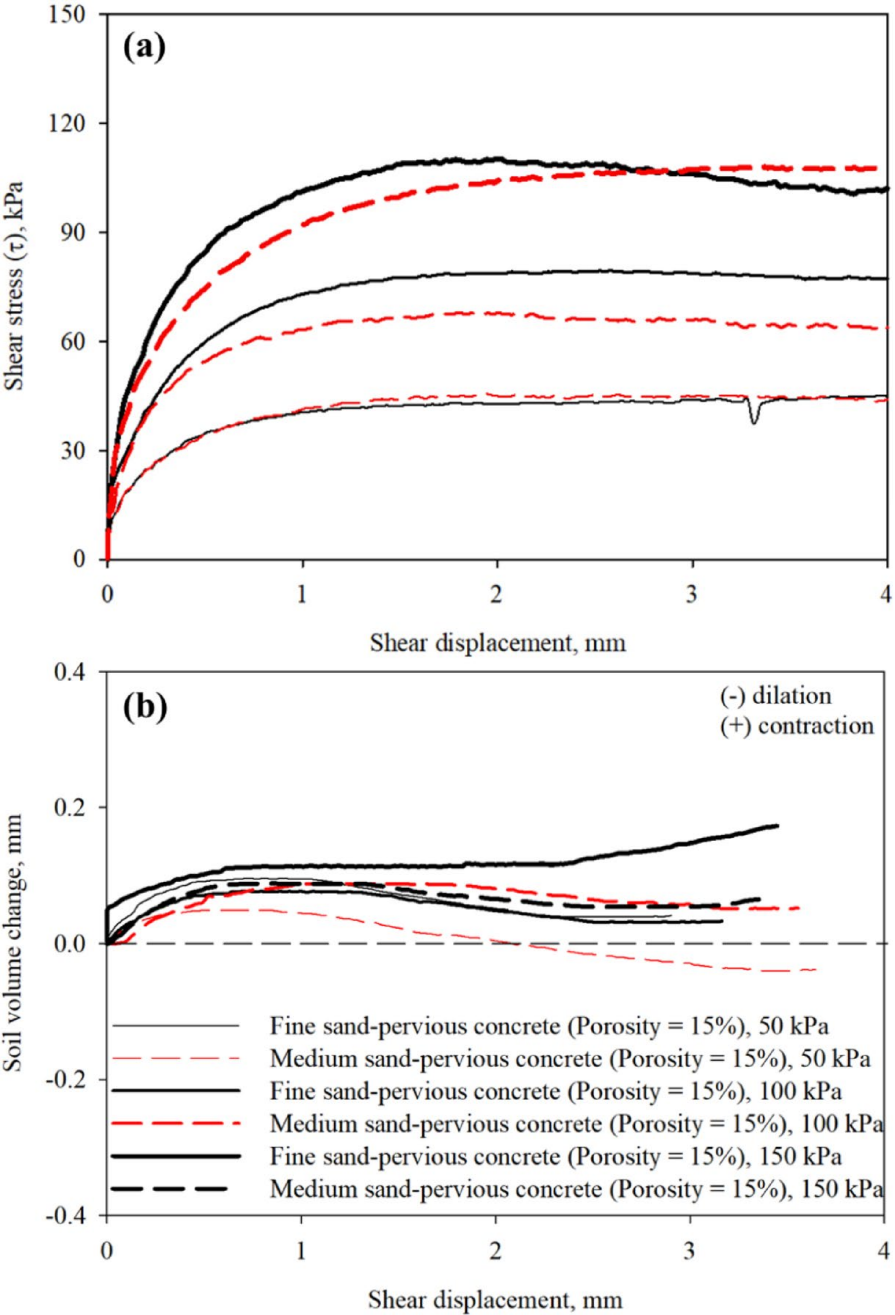
Results from interface shear tests on pervious concrete block with a porosity of 15% in fine and medium sands: (**a**) mobilized shear stress with shear displacement; and (**b**) soil volume change with shear displacement, at 50, 100, 150 kPa confining (normal) stress.

Figure 10a,b presents results from interface direct shear tests on poorly-graded fine and poorly-graded medium sands sheared against pervious concrete specimens with porosity of 15% at a constant shearing rate. Figure 10a shows the mobilized shear stress with shear displacement response curves for both fine and medium sand specimens at variable confining (normal) stress values of 50, 100, and 150 kPa. The soil volume change with shear displacement response curves for both sands are shown in Fig. 10b. As shown in Fig. 10a, the shear resistance at sand-pervious concrete of same surface roughness increases with confining (normal) stress level. The fine sand specimens mobilized larger interface shear resistance than medium sands for each confining (normal) stress of 50, 100, and 150 kPa.

The medium sand-pervious concrete surface interface exhibited dominant contractive behavior (except the test at 50 kPa), however, the fine sand-pervious concrete surface interface volume dilation with shear displacement (change from contraction to dilation after 1.5 mm), which results in larger interface shear resistance. In the case of poorly-graded fine sand, the roughness of surface allows the particles to entrap in the asperities (high level of random roughness) and causes more soil dilation and shear failure within soil mass (i.e., soil-soil failure), which was observed by Uesugi and Kishida^32^. In the case of poorly-graded medium sand (i.e., coarser sand), the particle size is larger and partially fills surface asperities which results in shear load transfer mechanism at soil-surface through sliding and interlocking with dominant contractive behavior, thus, slightly smaller interface shear resistance.

**Fig. 11:**
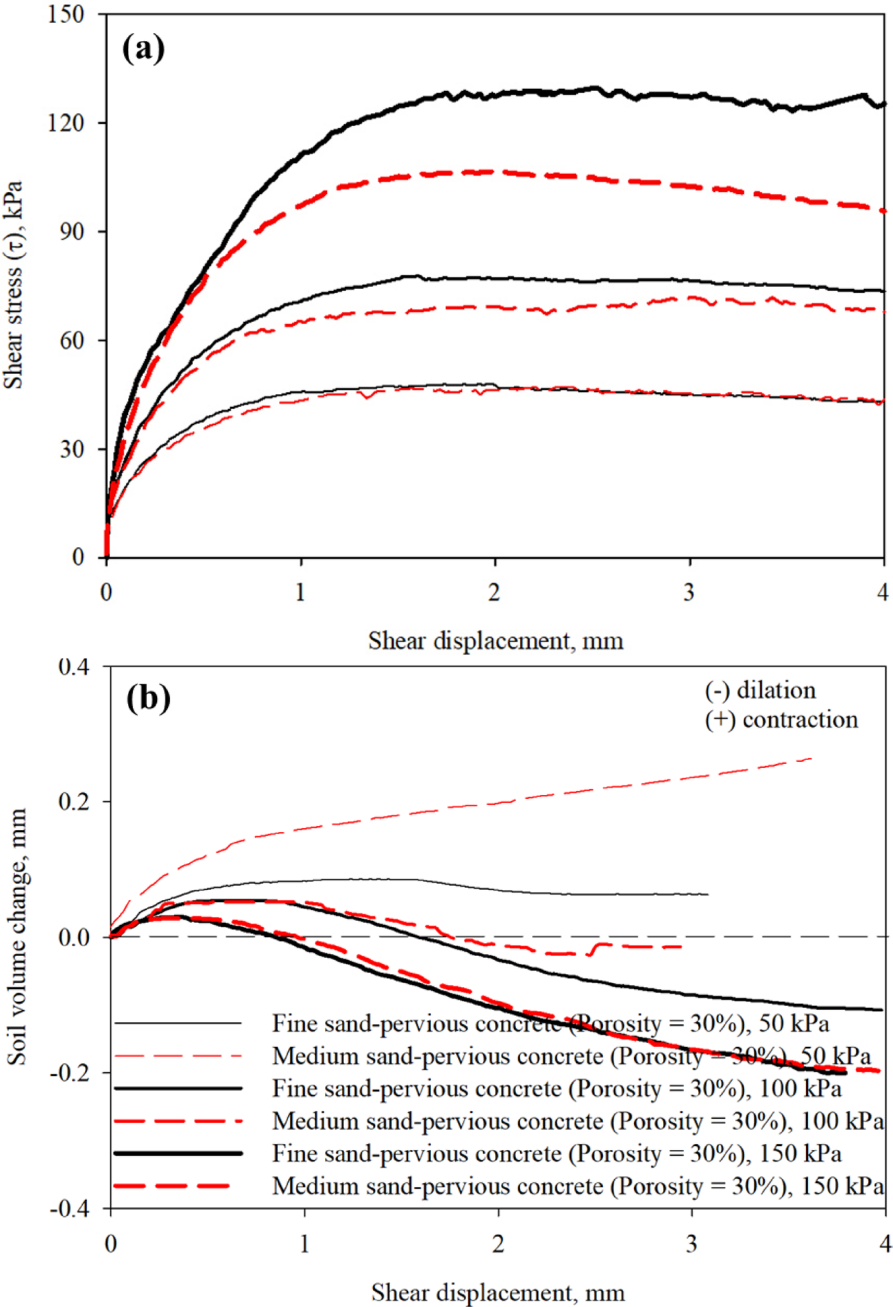
Results from interface shear tests on pervious concrete block with a porosity of 30% in fine and medium sands: (**a**) mobilized shear stress with shear displacement; and (**b**) soil volume change with shear displacement, 50, 100, 150 kPa confining (normal) stress.

Figure 11a,b presents results from interface direct shear tests on poorly-graded fine and poorly-graded medium sands sheared against pervious concrete specimens with porosity of 30% at a constant shearing rate. Figure 11a shows the mobilized shear stress with shear displacement response curves for both fine and medium sand specimens at confining (normal) stress values of 50, 100, and 150 kPa, respectively. The soil volume change with shear displacement response curves for both sands are shown in Fig. 11b. As shown in Fig. 11a, the shear resistance at sand-pervious concrete of same surface roughness increases with confining (normal) stress level. The fine sand specimens mobilized significantly larger interface shear resistance than medium sand for each confining (normal) stress of 50, 100, and 150 kPa. The poorly-graded fine sand-pervious concrete with 30% porosity interface showed a prevalent dilative behavior when compared to poorly-graded medium sand-pervious concrete with 30% porosity interface at the same confining (normal) stress. For fine sand, the high level of surface roughness allows soil particles to entrap and create a failure plane mainly through soil mass than sliding and interlocking as in the case of medium sand at the same value of confining (normal) stress. The transfer of shear stress between the fine and medium sands and pervious concrete surfaces mainly occur through friction (sliding) or/and interlocking accompanied by volume dilation leads to an increase in the confining (normal) stress applied on the soil specimen with shear displacement. Similar observation were made by other researchers from tests on rough smooth and rough surfaces simulate the surface condition of foundation elements in different type of soils^27,40,45,46,70,71^.

**Fig. 12:**
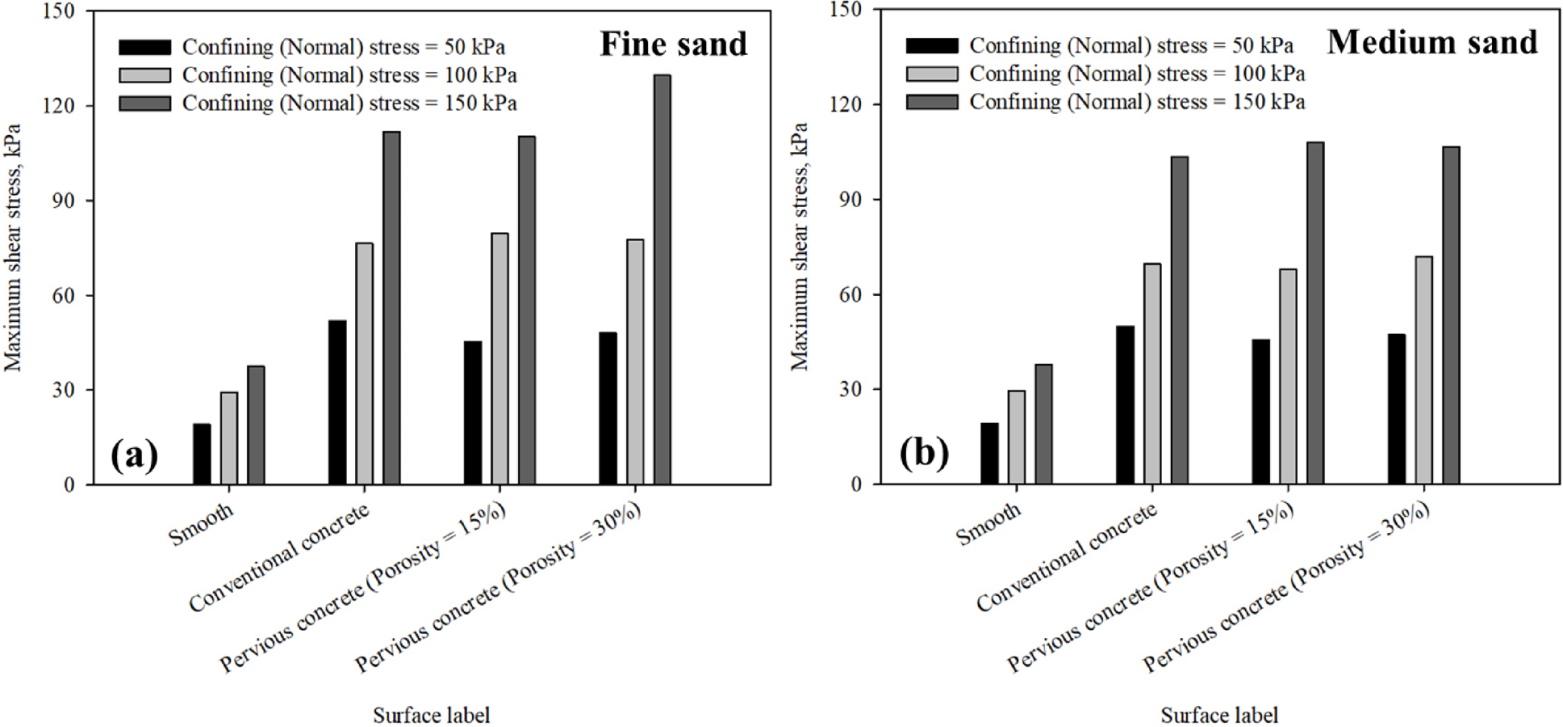
Maximum shear stress at the interface between all surfaces and (**a**) fine, and (**b**) medium sands, at 50, 100, 150 kPa confining (normal) stress.

Figure 12a,b summarizes the maximum interface shear stress mobilized at the interface between sand and all tested surfaces including smooth (untextured), conventional and pervious concrete surfaces. Figure 12 shows that for all surfaces in fine and medium sand, the maximum interface shear stress mobilized increases with the increase in confining (normal) stress. It is found that conventional concrete and pervious concrete with 15% and 30% porosity, representing medium level of random roughness, mobilized same maximum interface shear resistance at 50 and 100 kPa, however, a significant increase was observed at 150 kPa confining (normal) stress in the case of pervious concrete with 30% porosity when sheared against poorly-graded fine sand.

**Fig. 13:**
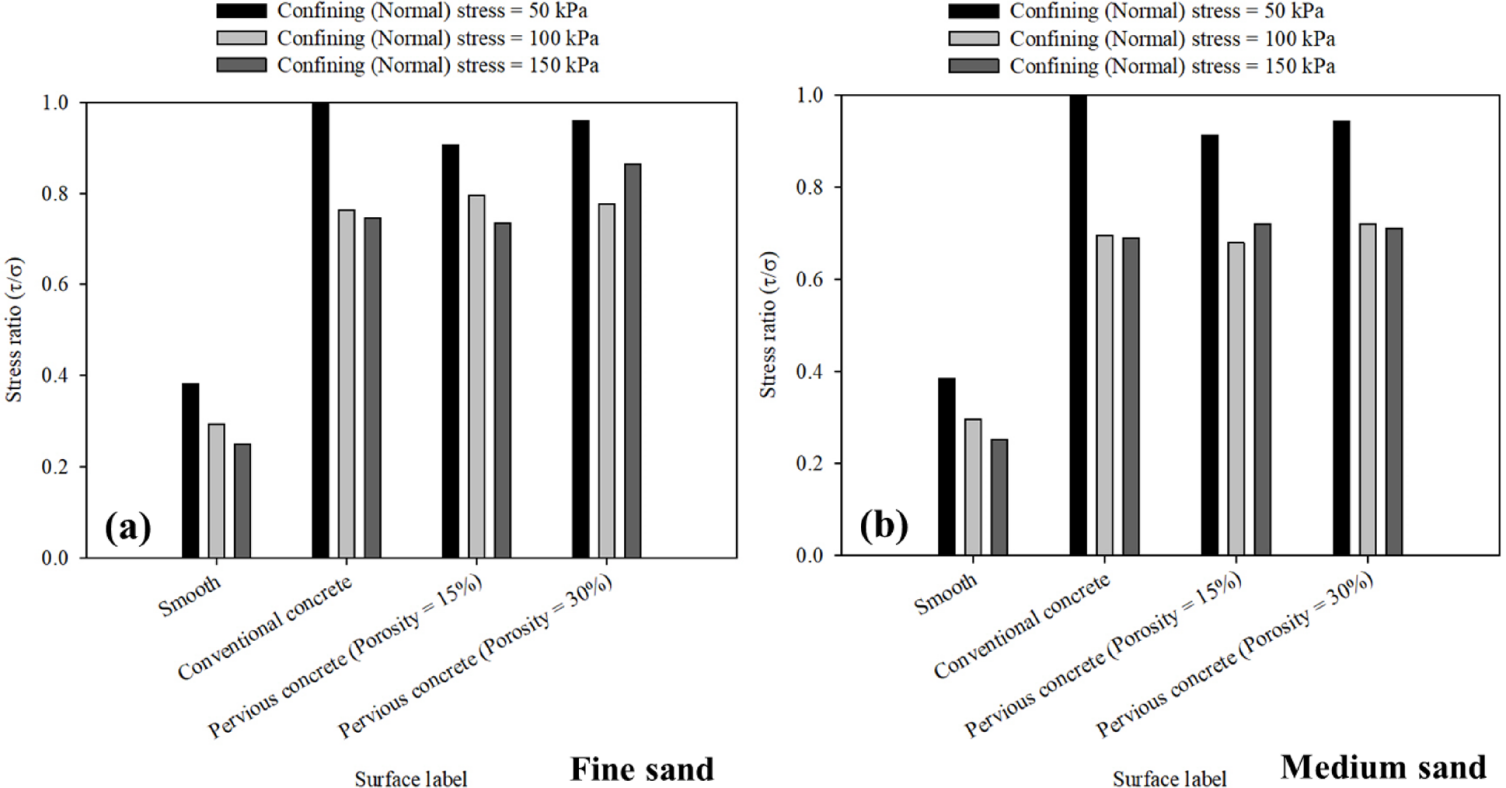
Maximum stress ratio at the interface between all surfaces and (**a**) fine, and (**b**) medium sands, at 50, 100, 150 kPa confining (normal) stress.

Figure 13a,b summarizes the maximum interface shear stress to confining (normal) stress (*τ*/*σ*) ratio mobilized at the interface between sand and all tested surfaces including smooth (untextured), conventional and pervious concrete surfaces. Figure 13 shows that for all surfaces in fine and medium sand, the maximum interface shear stress to confining (normal) stress (*τ*/*σ*) ratio mobilized decreases with the increase in confining (normal) stress. The maximum interface shear stress to confining (normal) stress (*τ*/*σ*) ratio for smooth surface ranged between 0.38 − 0.36 and 0.24–0.25 in fine and medium sands, respectively. For conventional concrete surface, the ratio ranged between 1.00 and 0.99 and 0.75 − 0.69 in fine and medium sands, respectively. While, for pervious concrete, it ranged between 0.91 − 0.90 and 0.73 − 0.72 in fine and medium sands, respectively for the case of 15% porosity surface, and ranged between 0.96 − 0.94 and 0.86 − 0.71 in fine and medium sands, respectively for the case of 30% porosity surface. A peak interface shear stress to confining (normal) stress (*τ*/*σ*) ratio in the range of 0.67 to 0.8 was reported by Zhang et al.^16^.

**Fig. 14:**
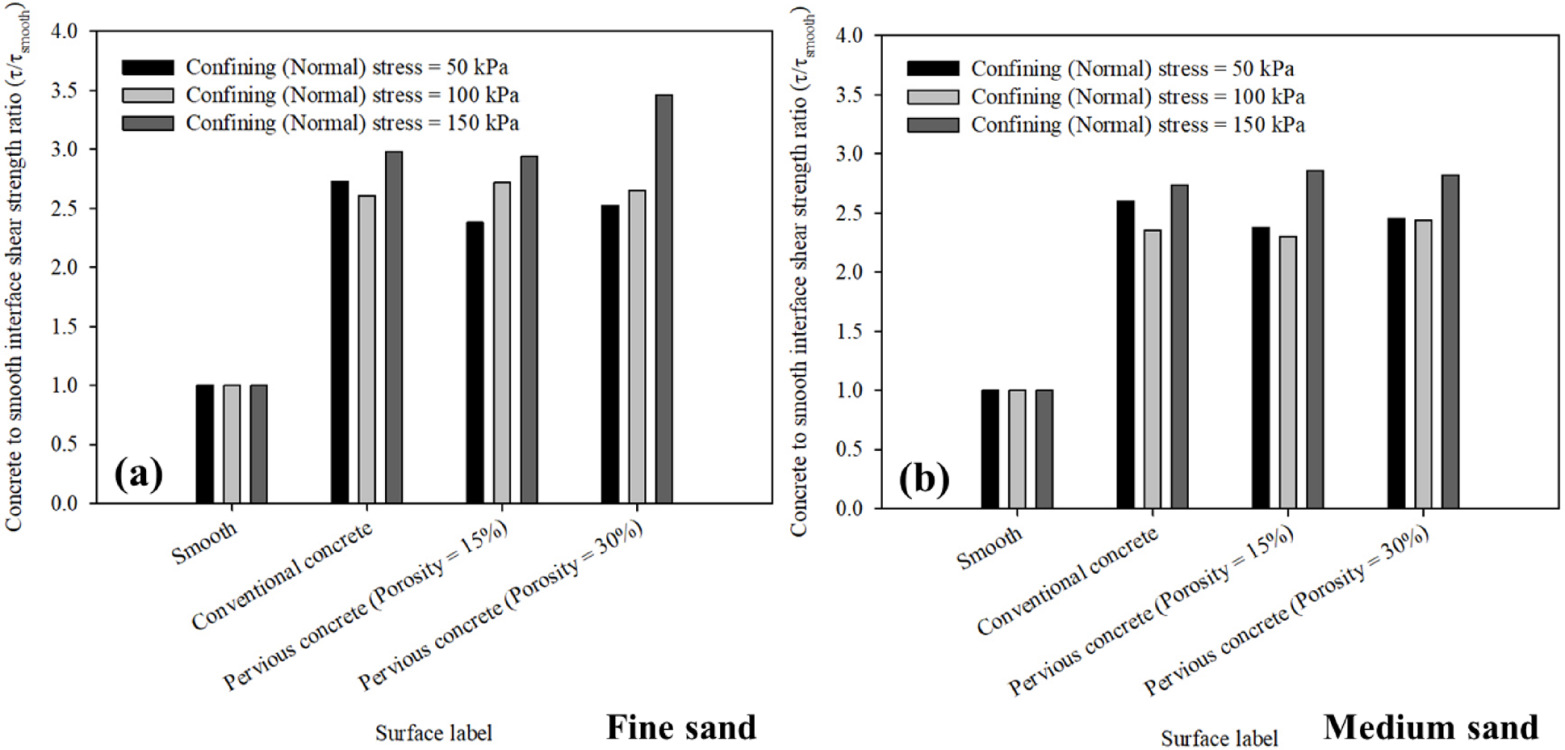
Concrete to smooth (untextured) shear strength ratio (*τ*/*τ*_smooth_) at the interface between all surfaces and (**a**) fine, and (**b**) medium sands, at 50, 100, 150 kPa confining (normal) stress.

As the smooth (untextured) surface was used to simulate the level roughness of smooth steel piles as the commonly used type of deep foundations in most geotechnical applications. Therefore, the maximum shear resistance mobilized at the interface between fine and medium sand against all surfaces was normalized to the shear resistance of fine and medium sand-smooth interface strength at confining (normal) stress of 50, 100, 150 kPa. Figure 14a,b summarizes the maximum concrete to smooth interface shear strength (*τ*/*τ*_smooth_) ratio mobilized at the interface between fine and medium sand and all tested surfaces including smooth (untextured), conventional and pervious concrete surfaces.

Figure 14 indicates that for all surfaces in fine and medium sand, the maximum concrete to smooth interface shear strength (*τ*/*τ*_smooth_) ratio increased with the increase in the surface roughness under different confining (normal) stress. The maximum concrete to smooth interface shear strength (*τ*/*τ*_smooth_) ratio for smooth surface was unity in fine and medium sands (smooth was used as a reference surface). The maximum concrete to smooth interface shear strength (*τ*/*τ*_smooth_) ratio for conventional concrete surface ranged between 2.73 and 2.60 and 2.98–2.73 in fine and medium sands, respectively. While it ranged between 2.38 and 2.36 and 2.94–2.85 in fine and medium sands, respectively for the case of 15% porosity surface and ranged between 2.52 and 2.45 and 3.46–2.82 in fine and medium sands, respectively for the case of 30% porosity surface. The results reported herein from interface direct shear tests on poorly-graded fine and medium sand against smooth (untextured), conventional concrete, pervious concrete with 15% and 30% confirm that the pervious concrete could yield high levels of interface strength due to its irregular surface roughness.

### Implications

The results presented herein indicate that the surface of pervious concrete piles with predetermined design porosity can mobilize interface shear resistance that is considerably larger than the resistance mobilized by smooth (untextured) surface or/and comparably larger than conventional concrete piles. Important implications arise from the fact that pervious concrete received an increasing interest in different engineering applications such as: (1) pervious concrete panels impregnated with phase change materials (PCMs) stored in porous aggregates for cooling/heating purposes^15^, (2) bio-grouted pervious concrete pile that utilizes useful bacteria exists in site soils to induce calcium carbonate (CaCO_3_) precipitation to cement soil particles, which is known as microbial-induced calcium precipitation (i.e., MICP)^12^, (3) possible use of pervious concrete to fabricate energy piles with or without phase change materials impregnated in porous aggregate as these piles transfer mechanical loads as well as exchange heat with the surrounding soil. Thus, the aim achieved herein is to highlight the importance of a more comprehensive understanding of the shear behavior of the interface between cohesionless soil and pervious concrete piles that can help in the design of geotechnical systems with enhanced load-carrying capacity to resist applied loads.

## Conclusions

This research paper presented the first study that investigates the shear stress transfer mechanism and the volumetric change behavior at the interface between poorly-graded fine and medium sand and pervious concrete surfaces of different surface roughness monotonic axial loading. The motivation of the present study is to improve the understanding of shear behavior at sand-pervious concrete interfaces as the pervious concrete is gaining an increasing interest in geotechnical engineering applications The experimental investigation presented herein consisted of a series of interface direct shear tests performed on specimens of poorly-graded fine and medium sands sheared against smooth (untextured), conventional concrete, and pervious concrete of different roughness and porosity utilizing a modified direct shear device. The shear resistance and volume change response at the interface was directly measured at different levels of confinement. The following conclusions can be made based on the study:

- Shearing smooth (untextured) surface against poorly-graded fine and medium sands mobilized lower interface shear resistance that increases with confining (normal) stress at same interface and loading conditions as the stress transfer mainly occurs through friction or surface sliding. The poorly-graded fine and medium sand-smooth interfaces exhibited prevalent contractive behavior with volume contraction increases with the increase in confining (normal) stress.
- Shearing conventional concrete surfaces with irregular surface roughness against poorly-graded fine and medium sands mobilized larger interface shear resistance compared to smooth (untextured) surface at same interface and loading conditions. The interface shear resistance increased by 175.8% and 198.0% compared to sand-smooth interface resistance in fine and medium sand, respectively. The fine sand-concrete surface interface exhibited more soil dilatation than medium sand-concrete surface interface at different confinement levels. The soil dilation observed forces more of a soil-soil failure, which results in a larger interface shear resistance, which is anticipated in most geotechnical systems made with conventional concrete.
- The surface roughness of pervious concrete piles/foundation elements can be varied and yet controlled through varying the design porosity of concrete to get better control over the predicted interface shear resistance against surrounding soil.
- The shear resistance and volume change responses at the interface between poorly-graded fine and medium sands is significantly influenced by surface roughness of pervious concrete surfaces as the roughness of pervious concrete is influenced by design porosity. The pervious concrete surfaces fabricated at porosity of 30%, with a higher level of roughness, mobilized a considerably higher interface shear resistance and volume dilation than the previous concrete surfaces with lower level of roughness (i.e., porosity of 15%), which experienced a prevalent higher resistance in poorly-graded fine sand than in medium sand. It was found that pervious concrete surfaces can achieve high levels of interface resistance compared to geotechnical systems with conventional surfaces. The shear resistance increased by 193.6% and 245.6% compared to sand-smooth interface resistance at porosity of 15% and 30%, respectively.

The current research provides baseline (reference) results to improve understanding of the behavior of sand-pervious concrete interface under monotonic loading. Further studies are required to investigate the behavior of bacteria cemented sand-pervious concrete interface under axial loading as well as the behavior of soil-pervious concrete energy pile interface under thermal loading.

## Data Availability

‘All data generated or analysed during this study are included in this published article’.
